# Supplementary material for: Adaptative machine vision with microsecond-level accurate perception beyond human retina
Source: Nat Commun. 2024 Jul 24;15:6261. doi: 10.1038/s41467-024-50488-6 (PMC11269608; doi:10.1038/s41467-024-50488-6)
Supplement: Supplementary file 1 — Supplementary Information [file 41467_2024_50488_MOESM1_ESM.docx]

Supplementary Information

**Adaptative machine vision with microsecond-level accurate perception beyond human retina**

Ling Li^1^, Shasha Li^2^, Wenhai Wang^1^, Jielian Zhang^1^, Yiming Sun^1^, Qunrui Deng^1^, Tao Zheng^1^, Jianting Lu^3^, Wei Gao^1^, Mengmeng Yang^1^, Hanyu Wang^1^, Yuan Pan^1^, Xueting Liu^1^, Yani Yang^1^, Jingbo Li^4,5^, Nengjie Huo^1,5*^

*^1^School of Semiconductor Science and Technology, South China Normal University, Foshan 528225, P.R. China*

*^2^School of Electronic Engineering, Chaohu University, Hefei 238000, China.*

*^3^National Key Laboratory of Science and Technology on Reliability Physics and Application of Electronic Component, China Electronic Product Reliability and Environmental Testing Research Institute, Guangzhou 510610, China.*

*^4^College of Optical Science and Engineering, Zhejiang University, Hangzhou 310027, China*

*^5^Guangdong Provincial Key Laboratory of Chip and Integration Technology, Guangzhou 510631, P.R. China.*

*Corresponding author

E-mail: njhuo@m.scnu.edu.cn


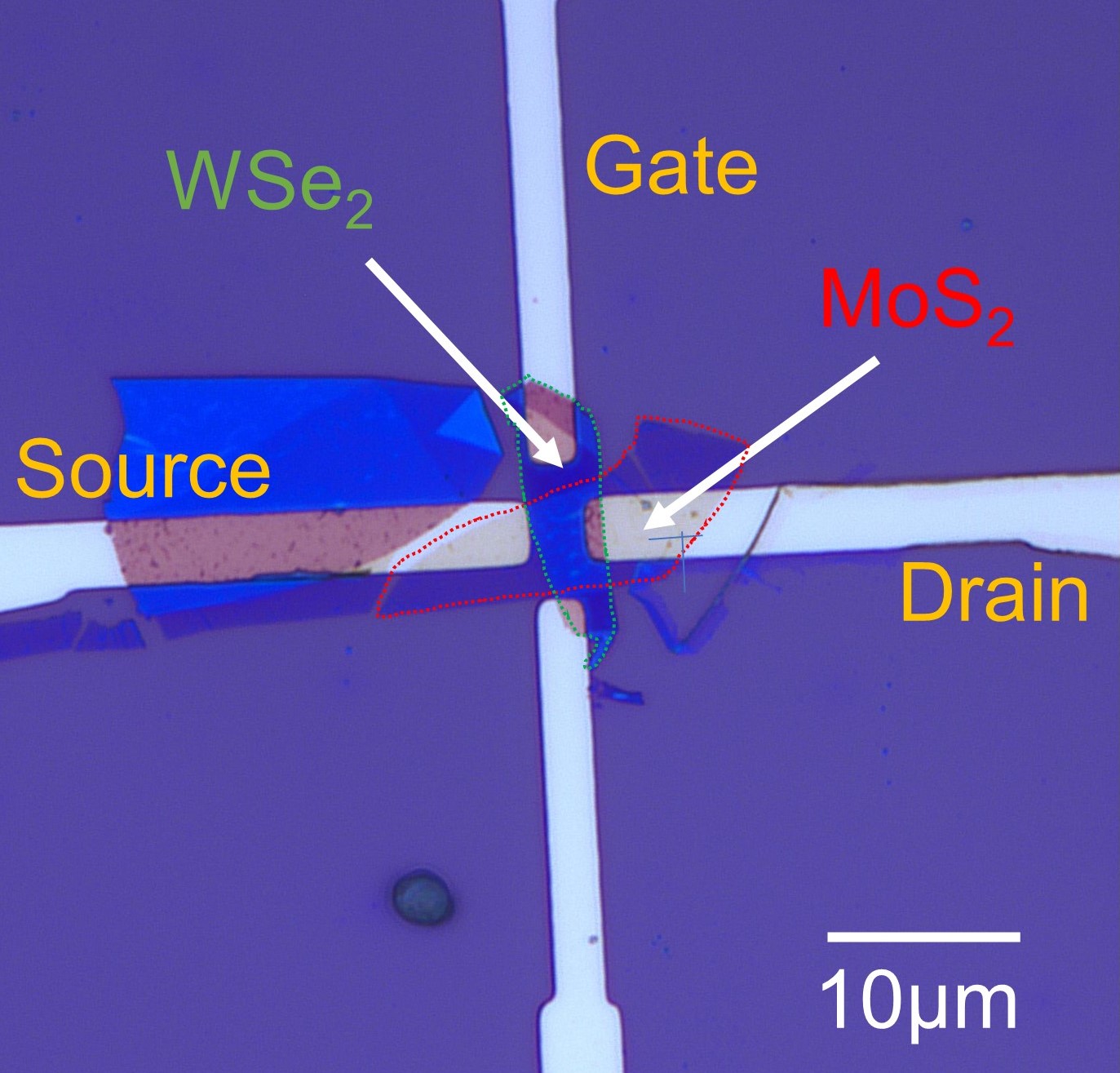


**Supplementary Figure 1.** Optical microscopy images of the device structure and electrical connections for the junction filed effect transistor (JFET) consisting of an ultrathin MoS_2_ transport channel and top WSe_2_ gate.


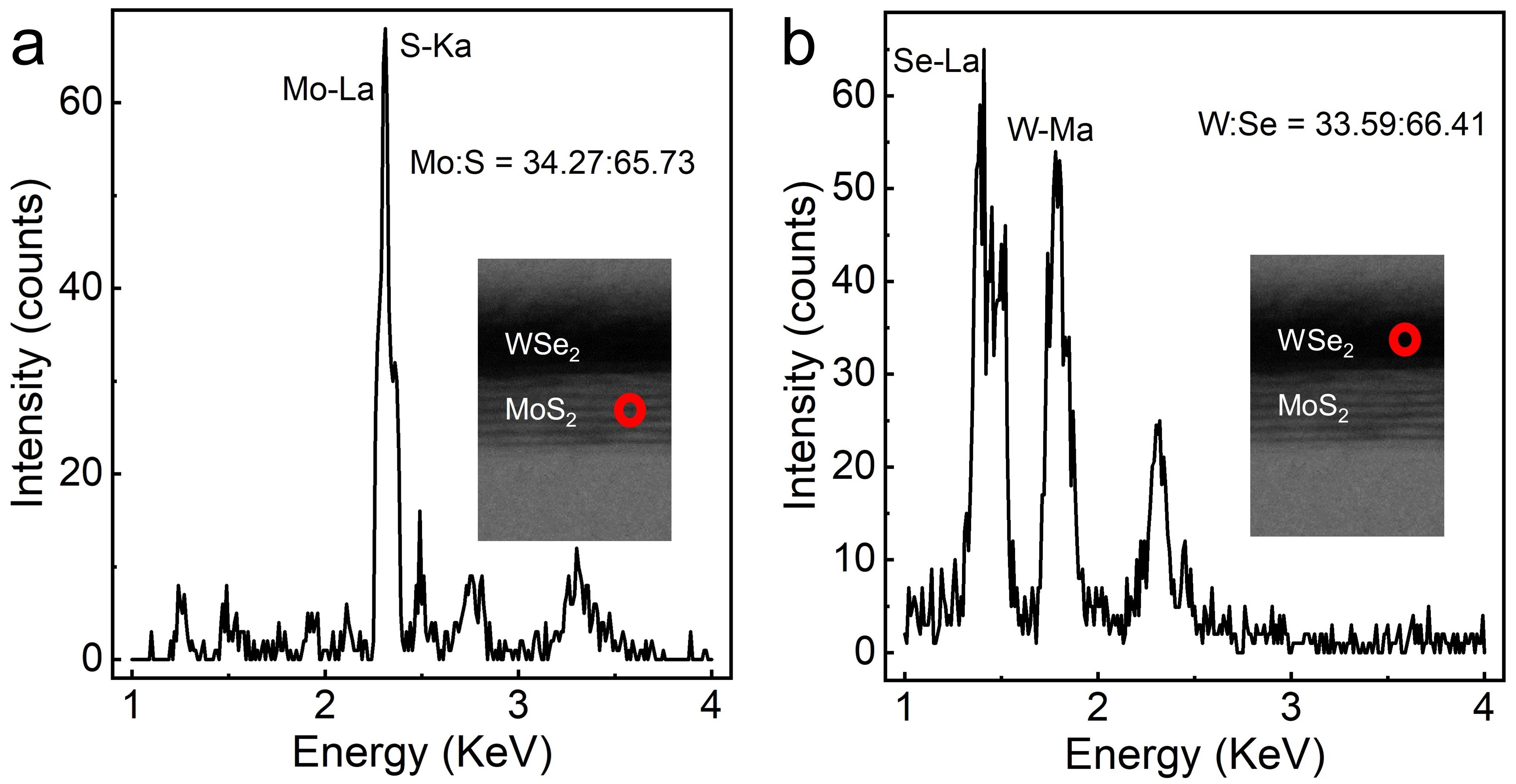


**Supplementary Figure 2.** EDX analysis of cross sectional MoS_2_/WSe_2_ interface, corresponding to (a) MoS_2_ and (b) WSe_2_, respectively.


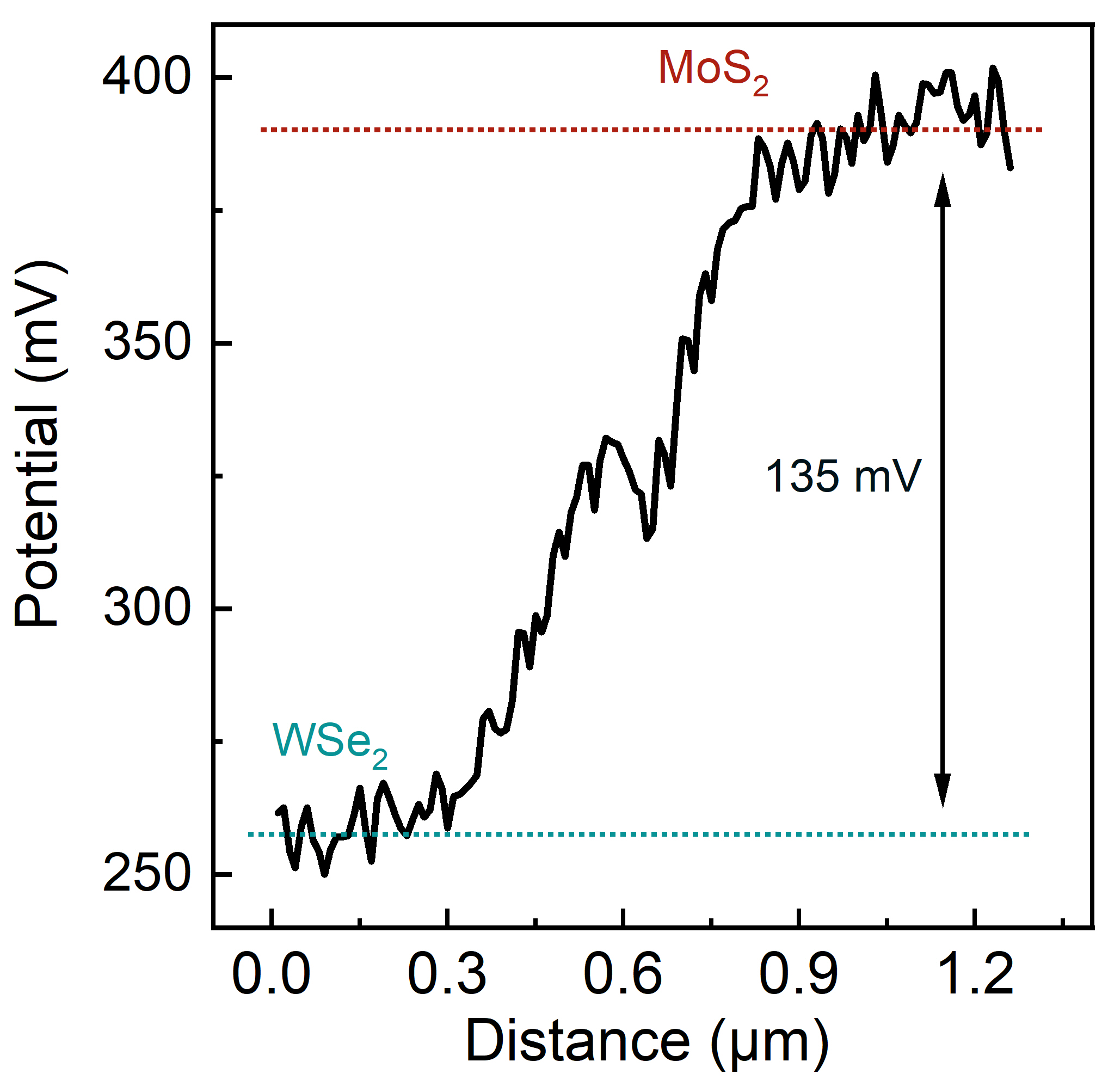


**Supplementary Figure 3.** In situ surface potential difference (SPD) plot of the measured MoS_2_/WSe_2_ interface.


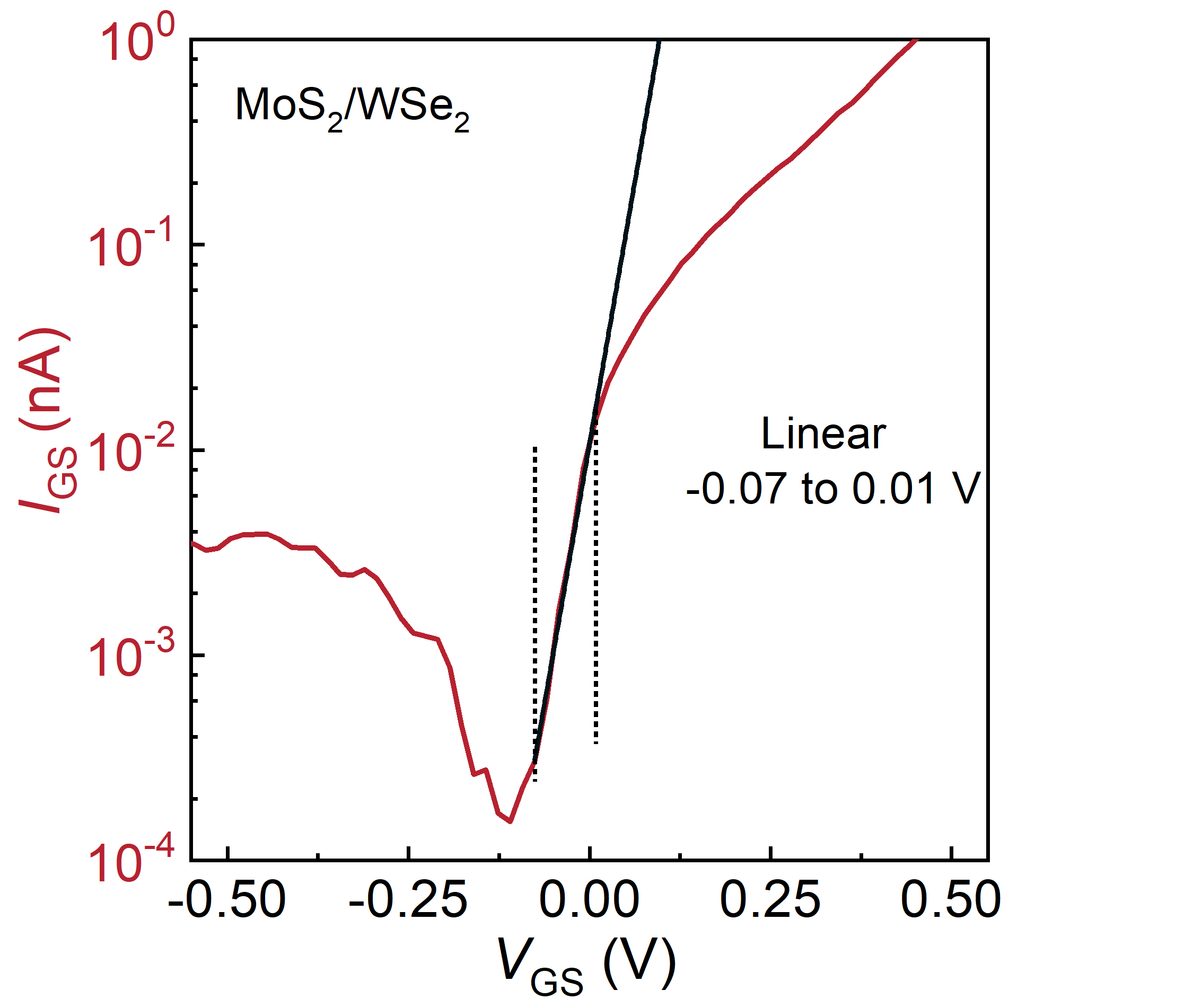


**Supplementary Figure 4.** The linear part of the *I-V* curve. The ideal factor (*n*) is calculated using the formula: $\text{n=}\frac{\text{q}}{\text{k}_{\text{b}}\text{T}}\frac{\text{dV}}{\text{dlnI}}$, where *q*, *k_b_* and *T* are elementary charge, Boltzmann constant and Kelvin temperature.


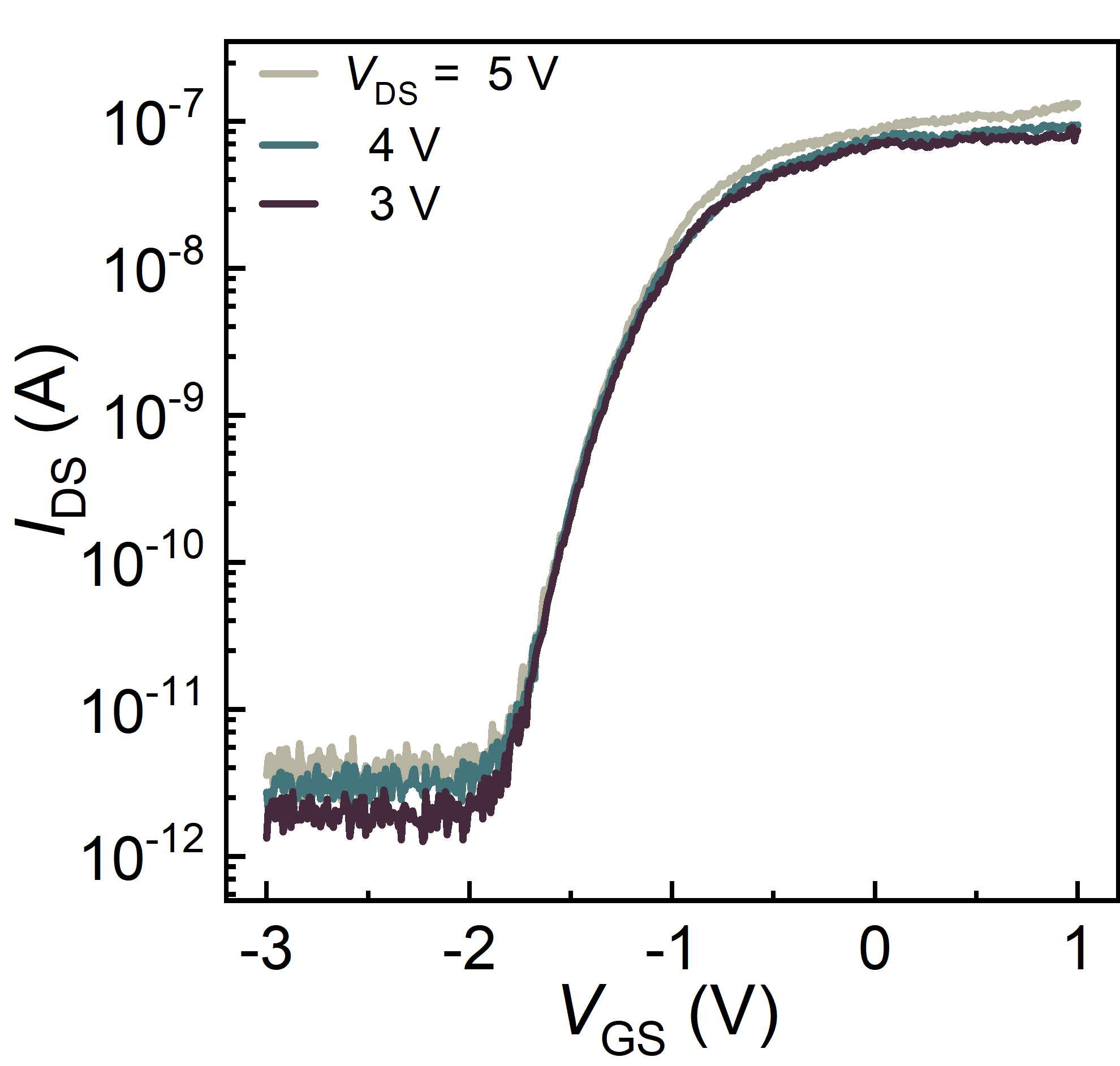


**Supplementary Figure 5.** Transfer characteristics of the device acting JFET. The transfer curves at *V*_DS_ at 3, 4 and 5 V are nearly overlapped, showing the device enters the saturation region from 3 to 5 V.


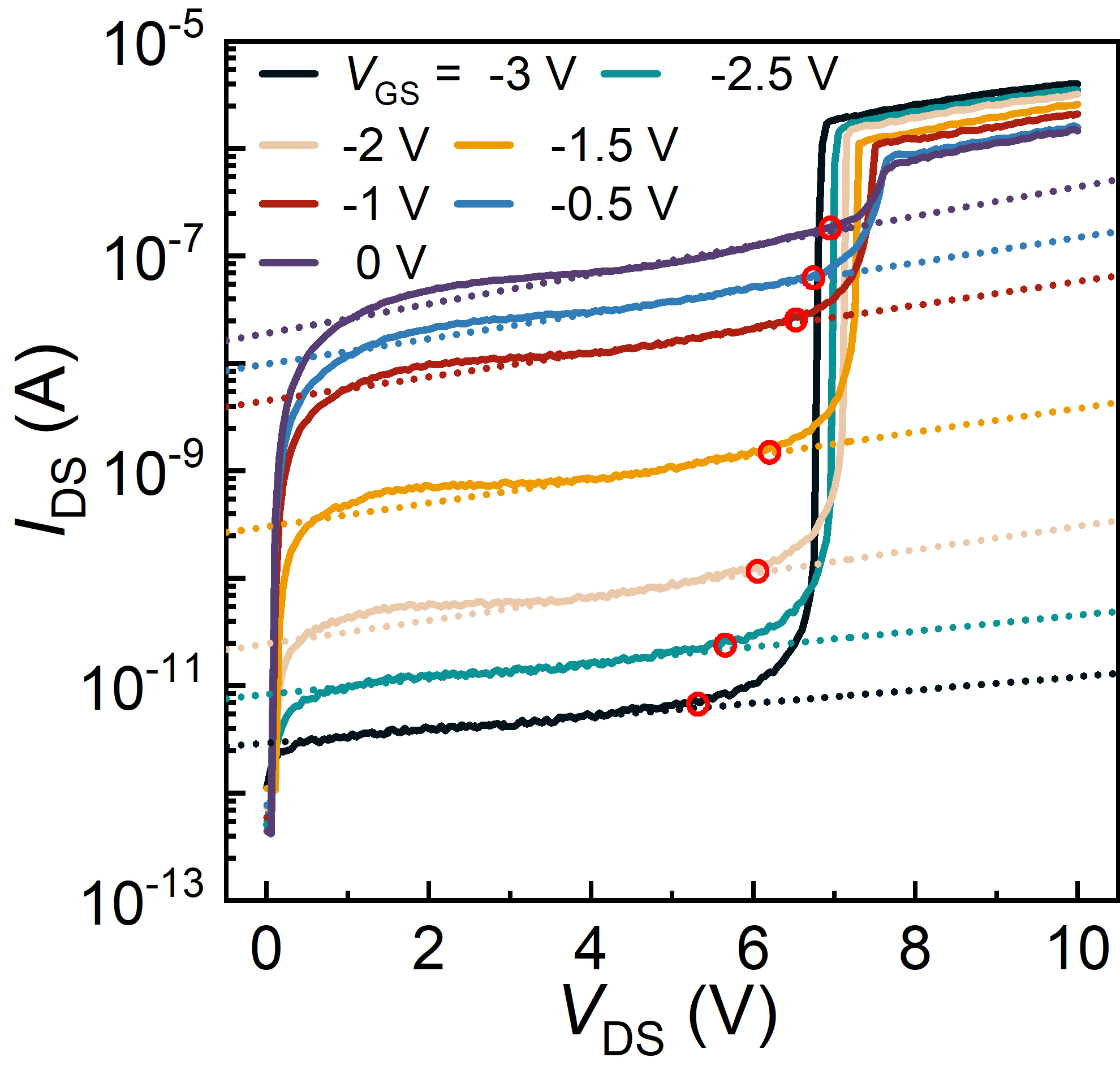


**Supplementary Figure 6.** The definition of the *V*_EB_ at different gate voltage (*V*_GS_). The *V*_EB_ is the intersection of the extension of the linear region of the saturation current and the *I*_DS_.


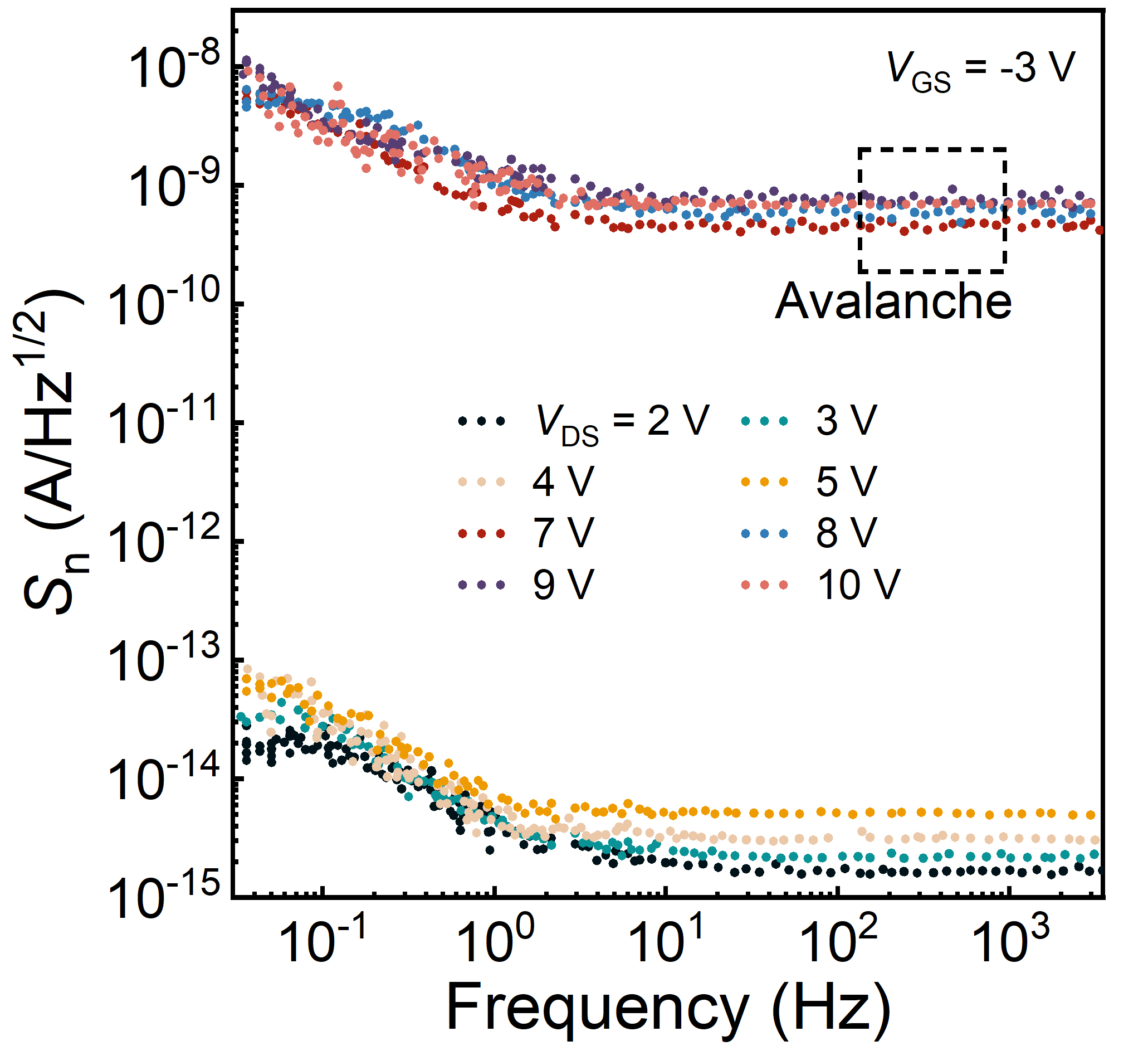


**Supplementary Figure 7.** Noise spectral density (*S*_n_) of the device at different *V*_DS_ by the Fourier transformation of dark current traces.


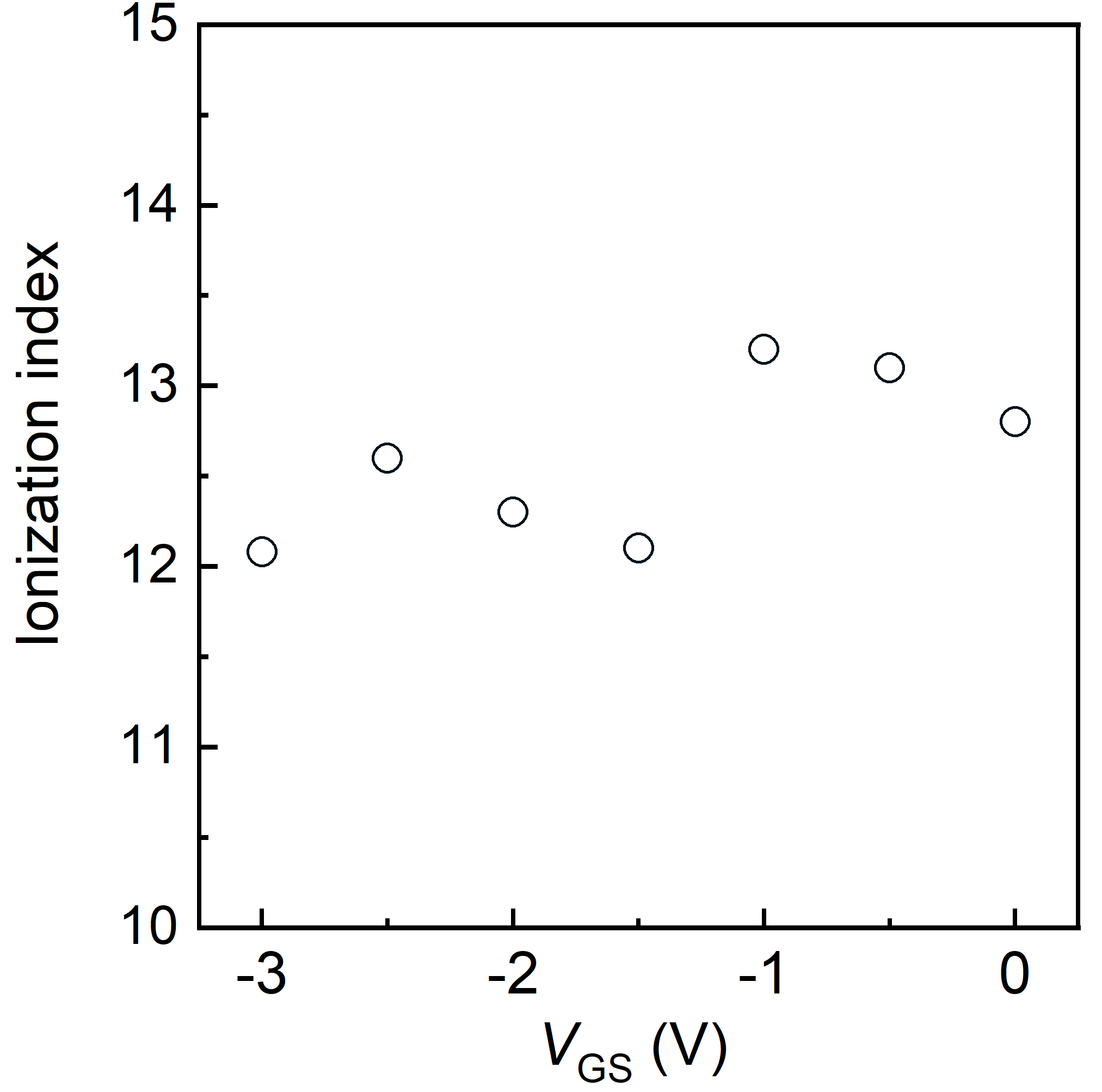


**Supplementary Figure 8.** The ionization index at different *V*_GS_.


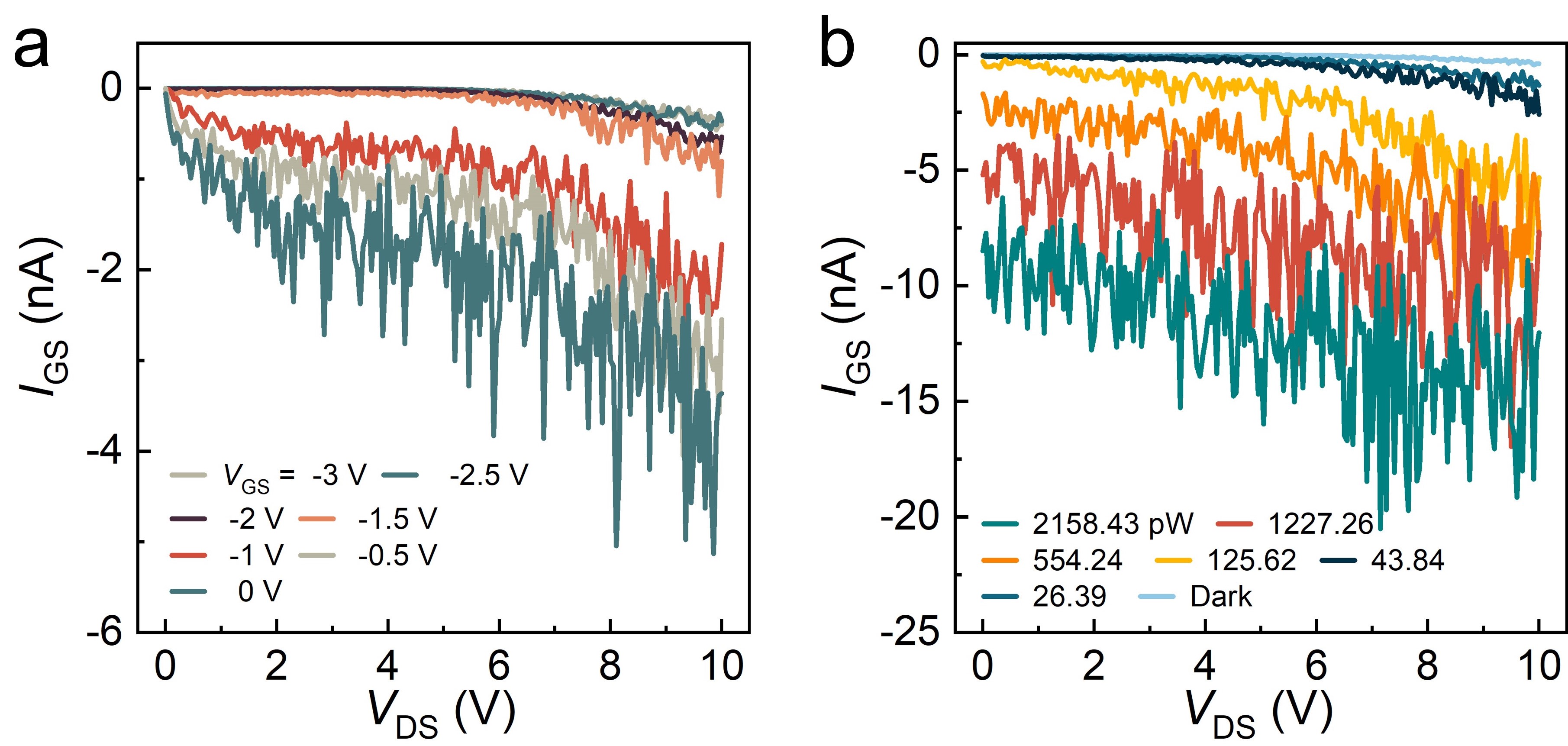


**Supplementary Figure 9.** The leakage current as a function of bias under (a) dark and (b) illumination.


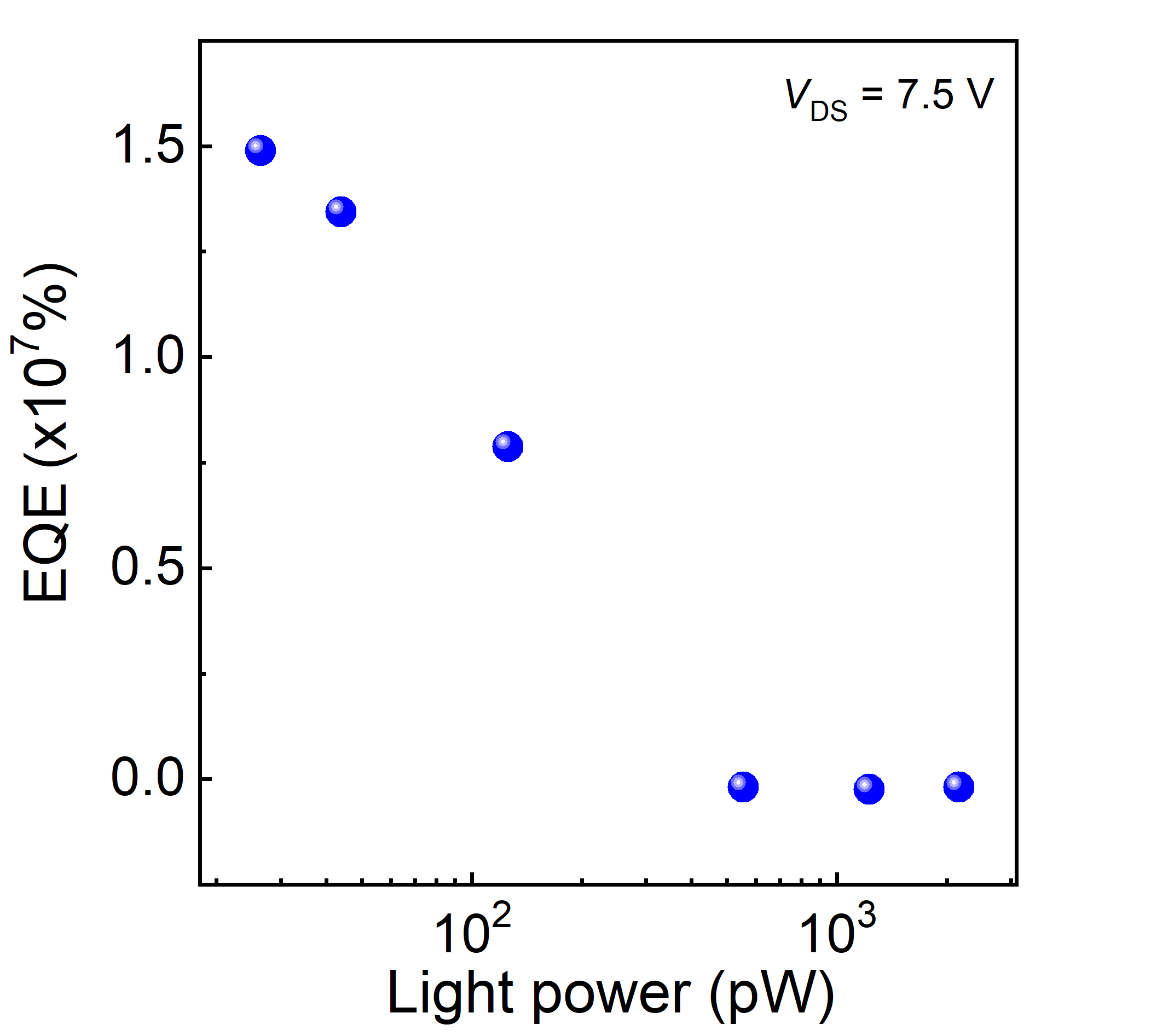


**Supplementary Figure 10.** Avalanche external quantum efficiency (EQE) at *V*_DS_ = 7.5 V as function of light power.


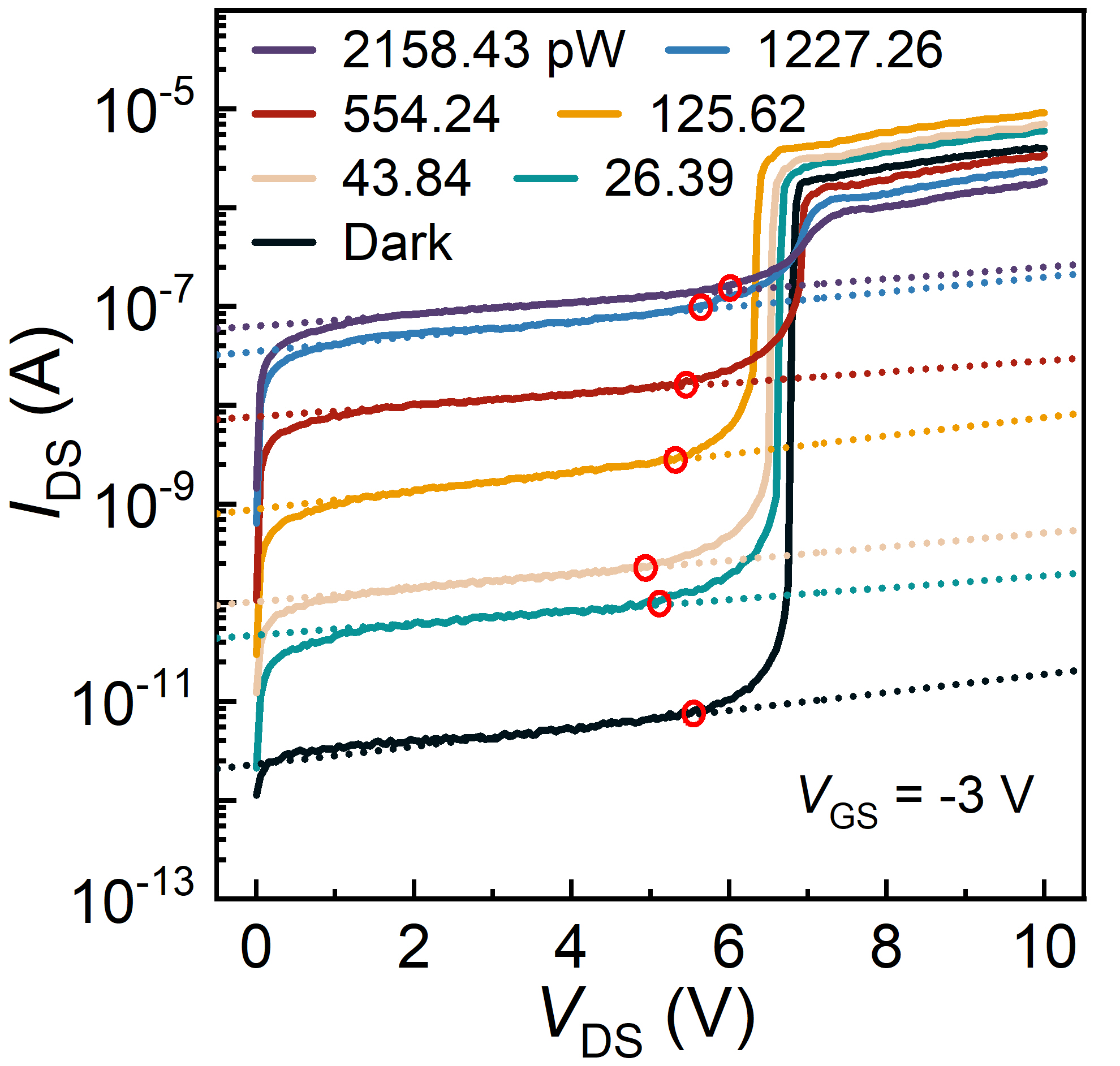


**Supplementary Figure 11.** The definition of the *V*_EB_ under different fixed laser power. The *V*_EB_ is the intersection of the extension of the linear region of the saturation current and the *I*_DS_.


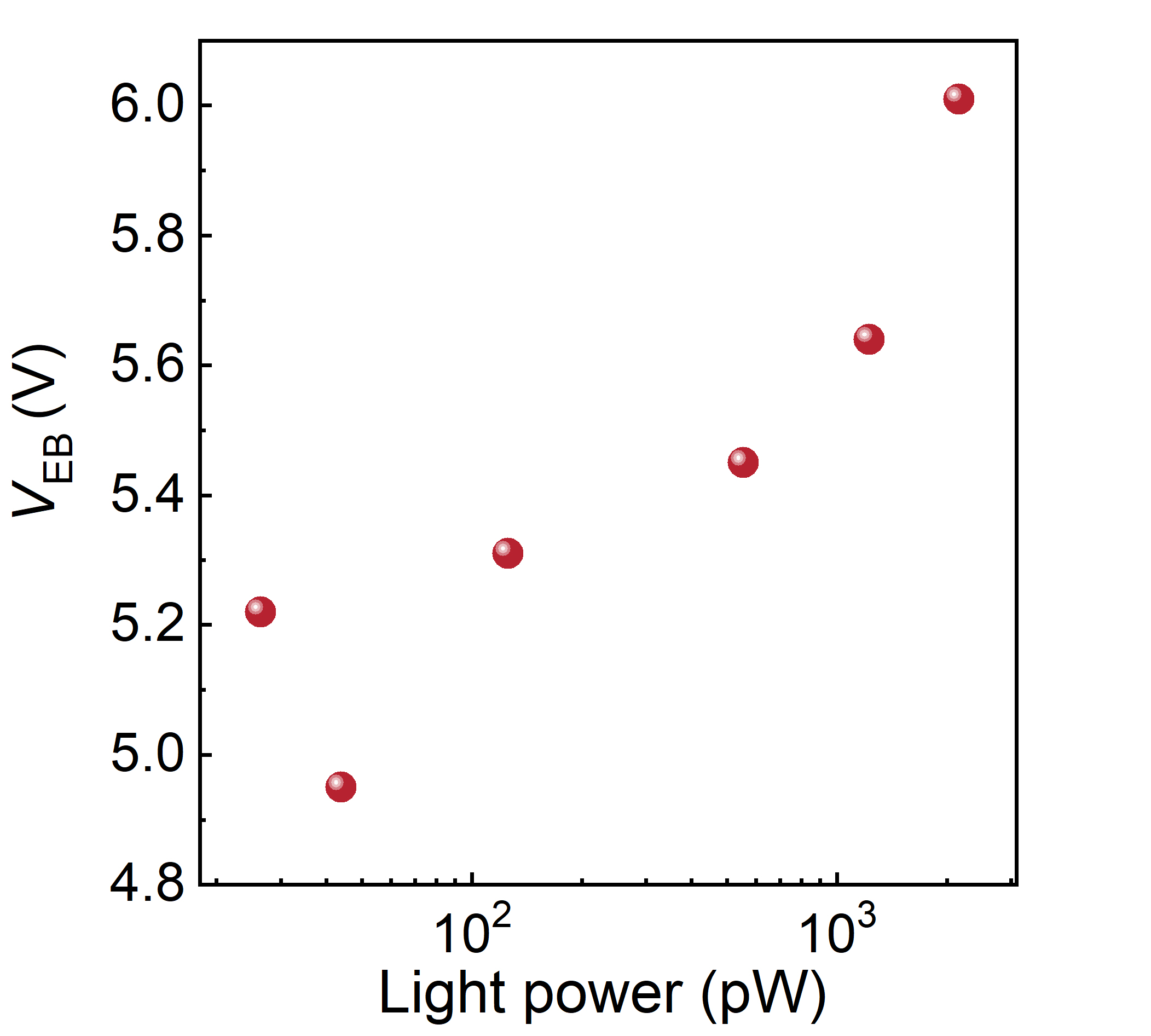


**Supplementary Figure 12.** The *V*_EB_ as function of laser power.


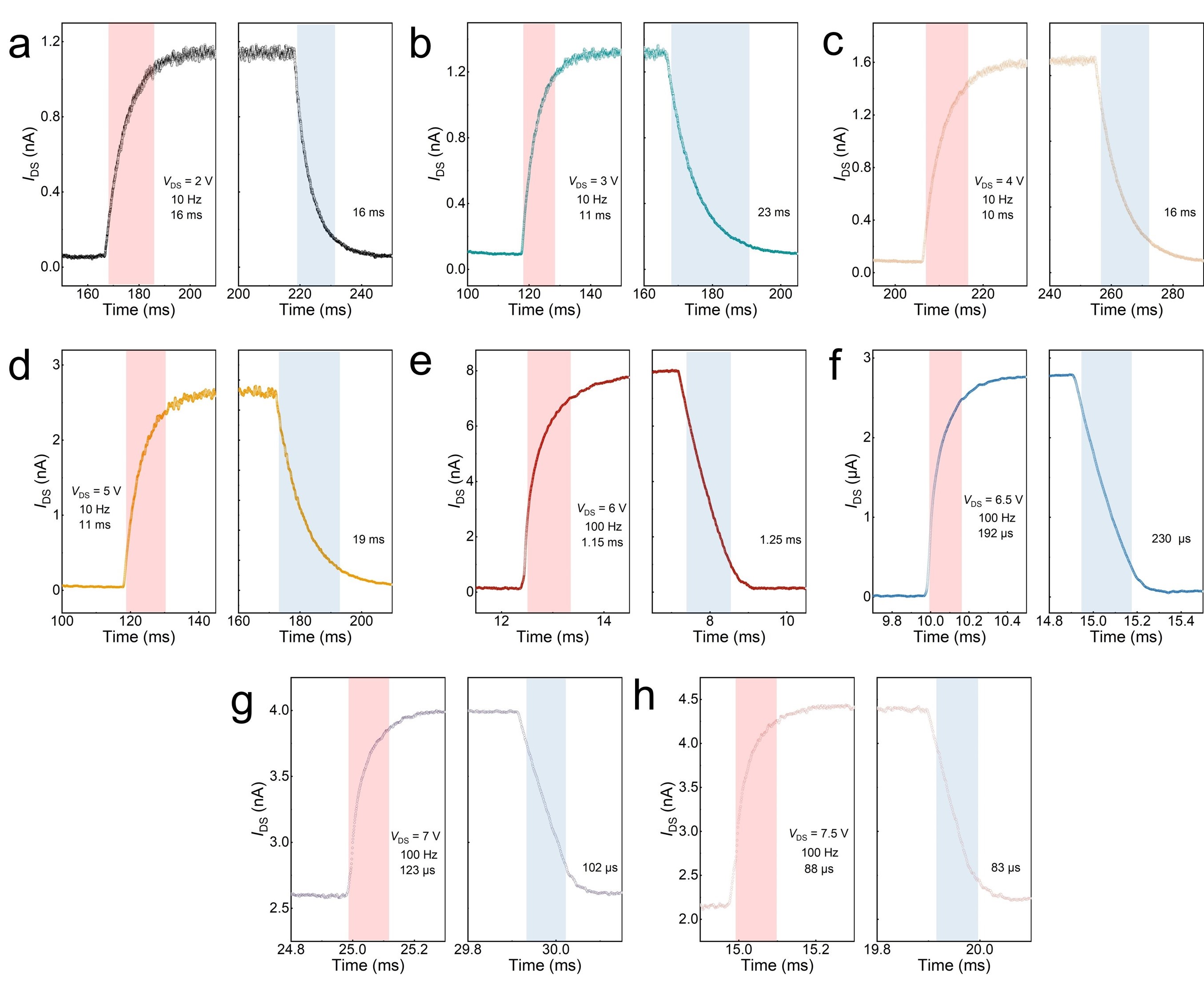


**Supplementary Figure 13.** Photo-response of the device at different *V*_DS_ under light power of 125.62 pW.


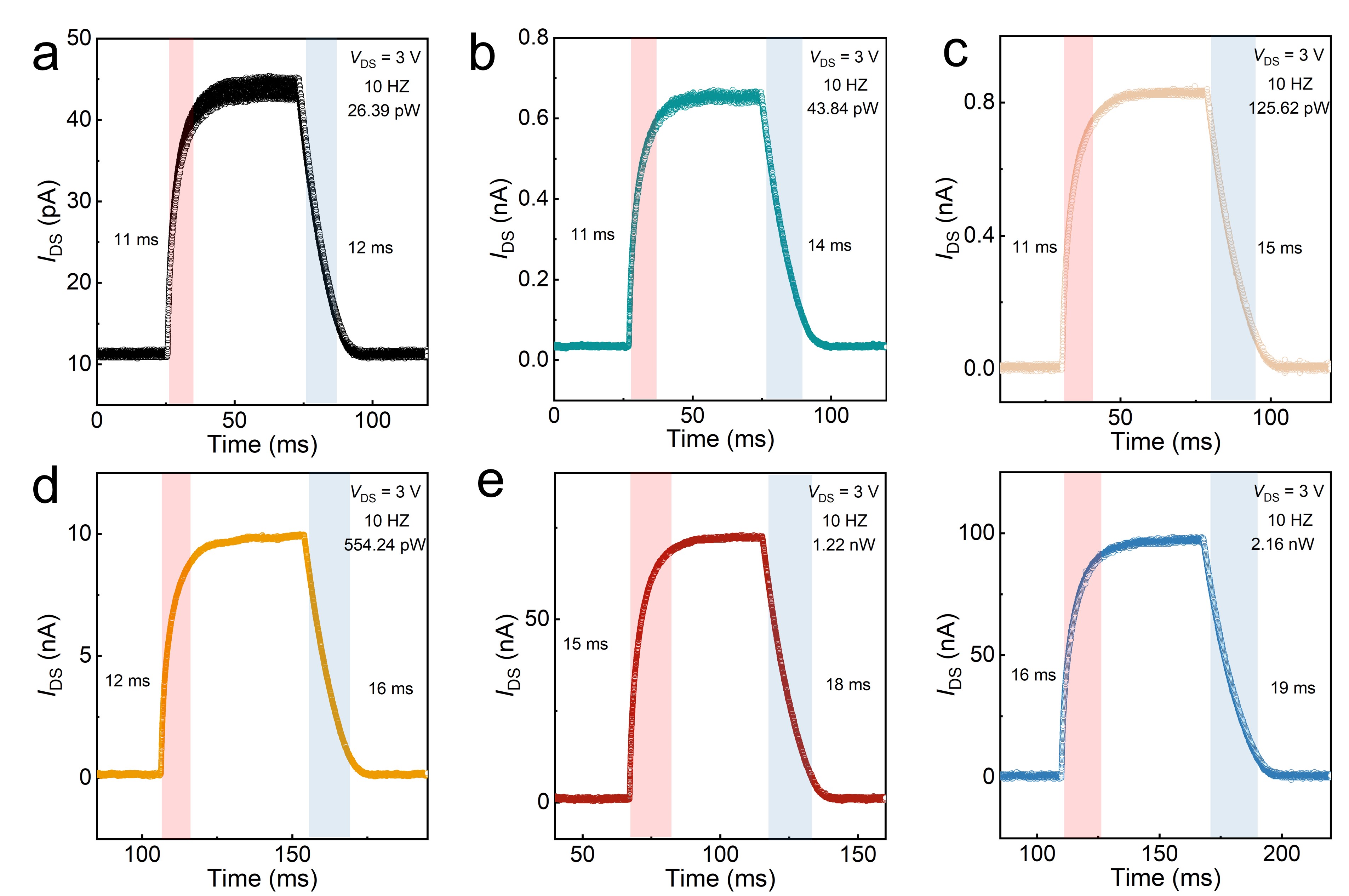


**Supplementary Figure 14.** Photo-response at *V*_DS_ = 3 V under different fixed light power.


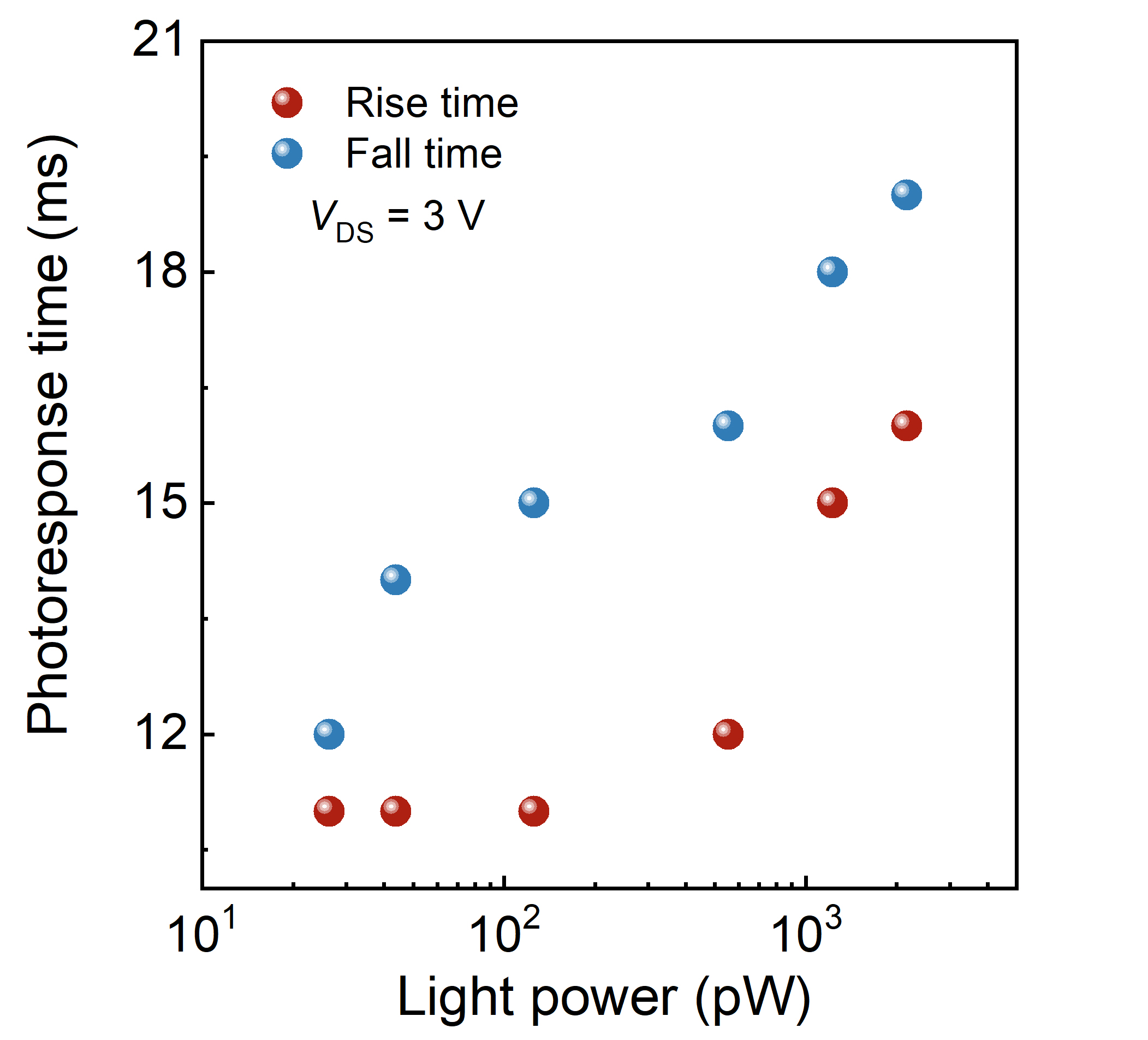


**Supplementary Figure 15.** Photo-response as function of light power at *V*_DS_ = 3 V.


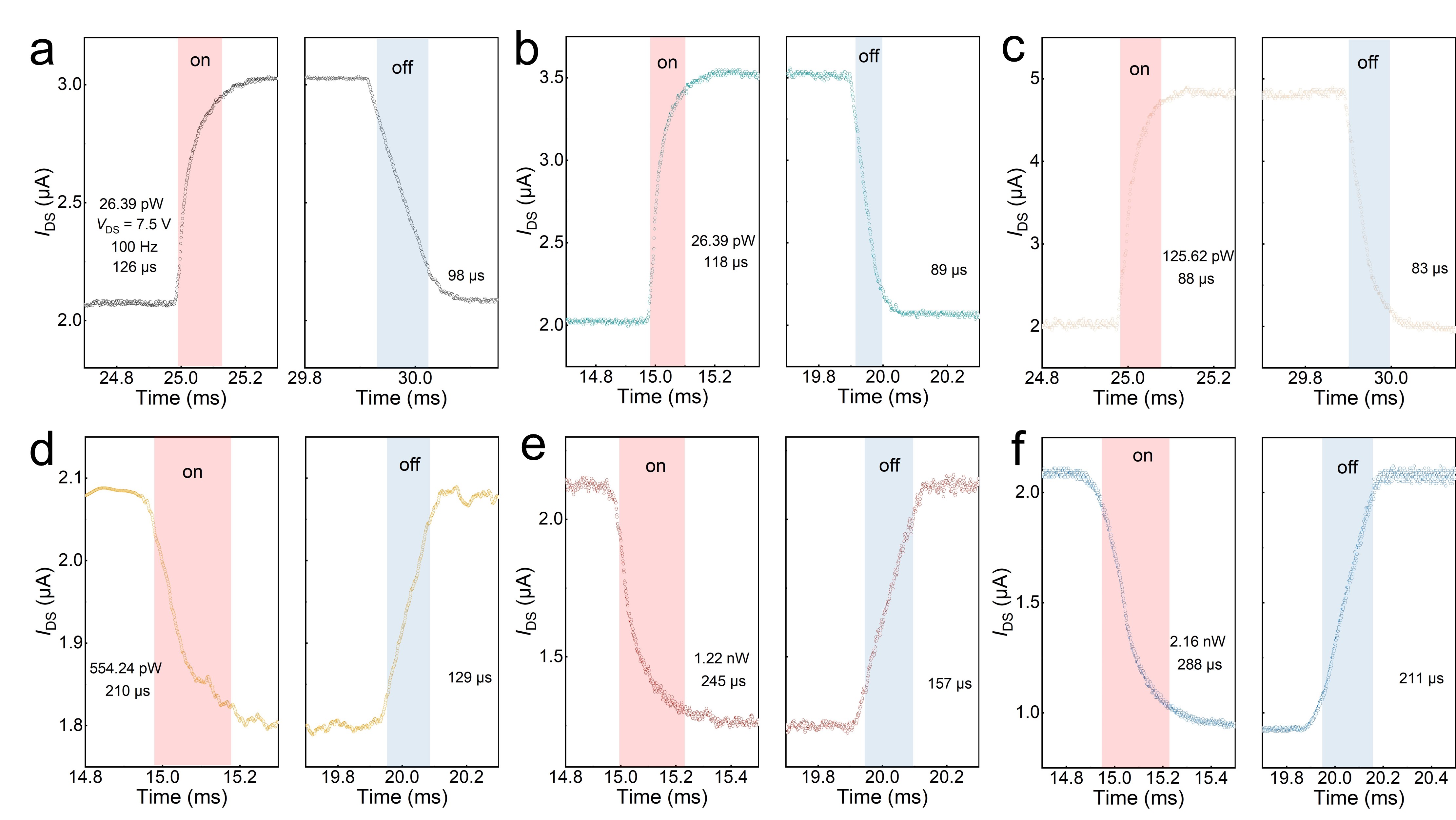


**Supplementary Figure 16.** Photo-response at *V*_DS_ = 7.5 V under different fixed light power.


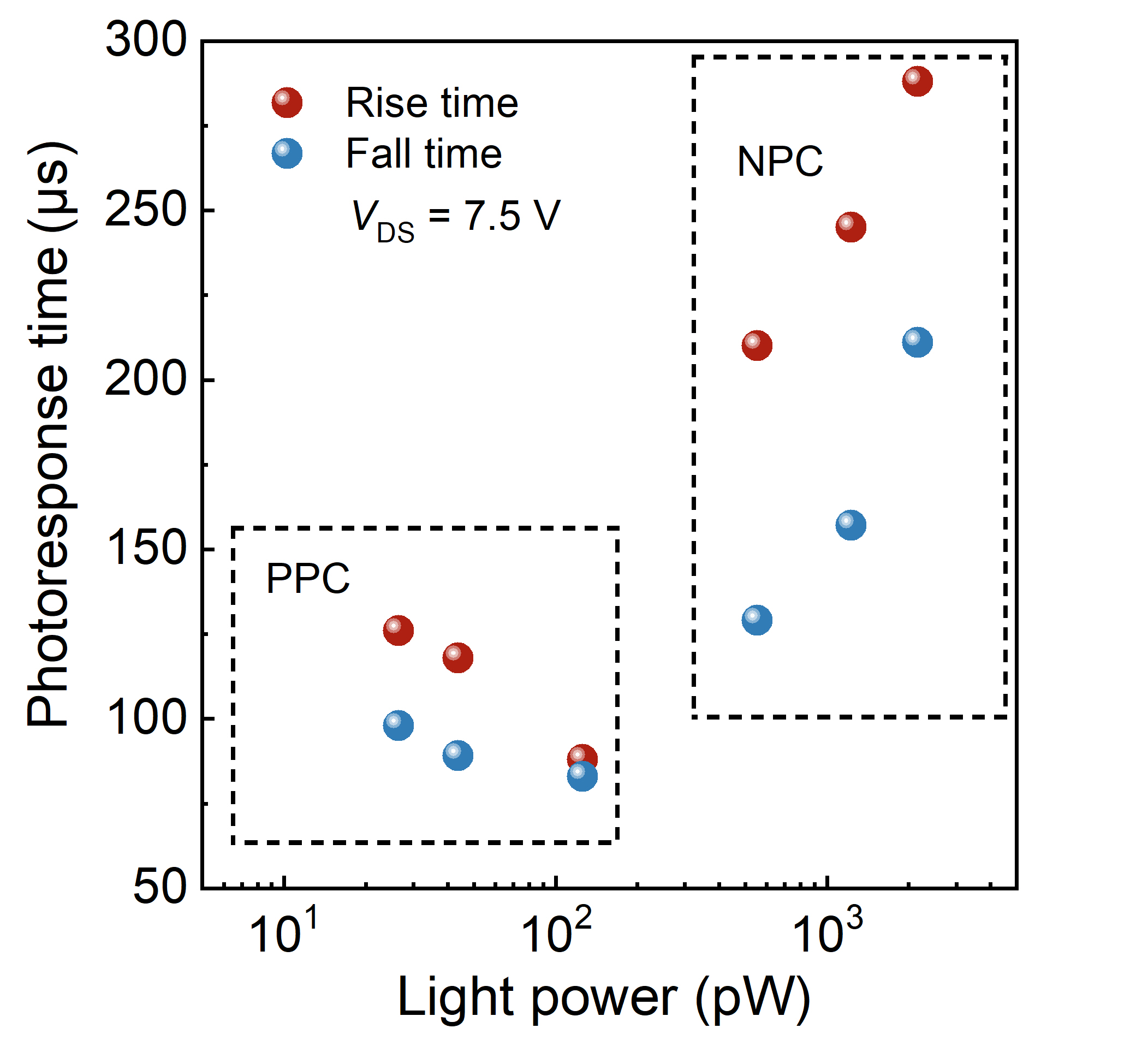


**Supplementary Figure 17.** Photo-response as function of light power at *V*_DS_ = 7.5 V, showing the PPC and NPC effect at weak and strong laser illumination, respectively.


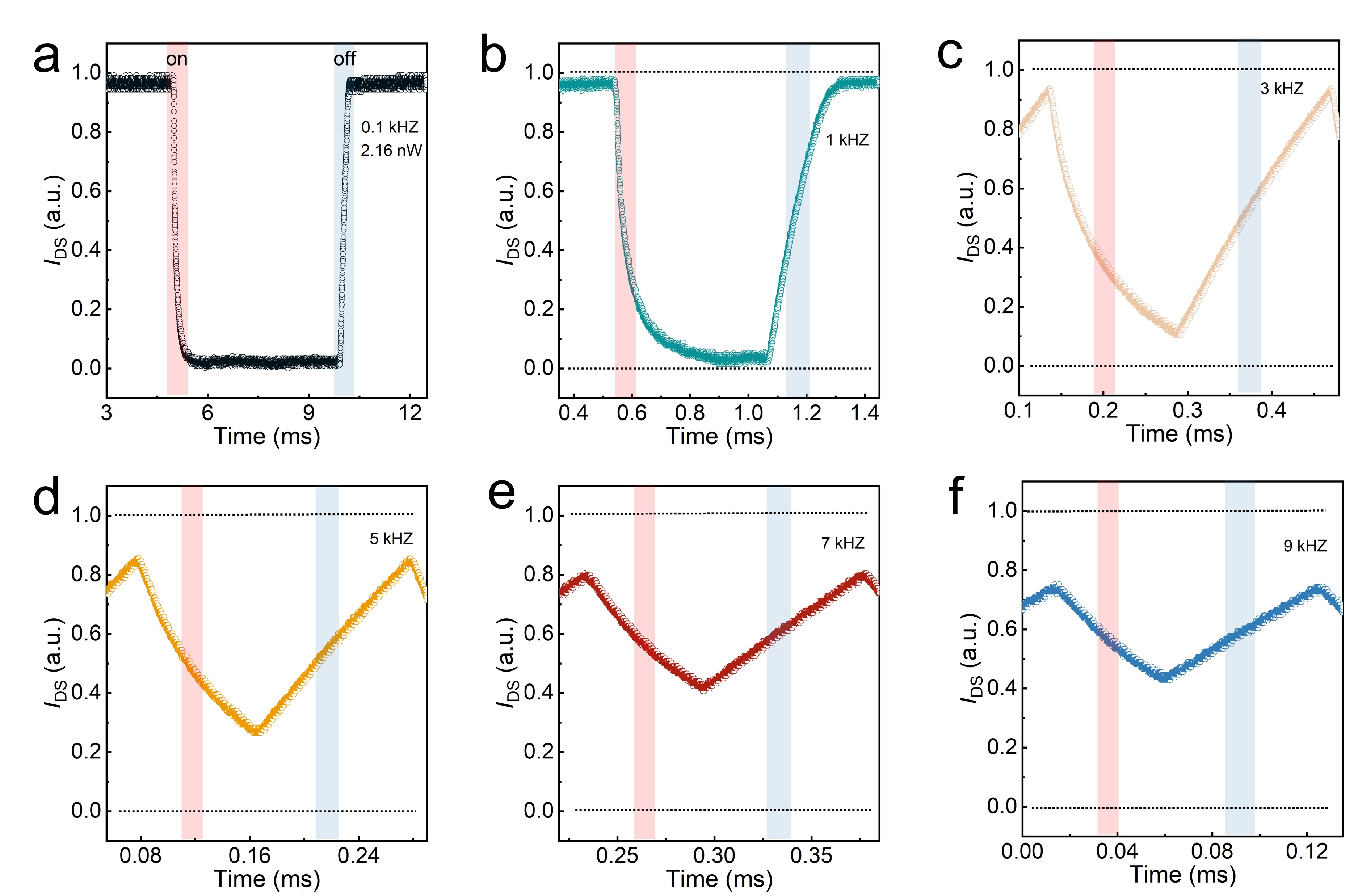


**Supplementary Figure 18.** Photo-response with different frequency at *V*_DS_ = 7.5 V and laser power of 2158.43 pW.


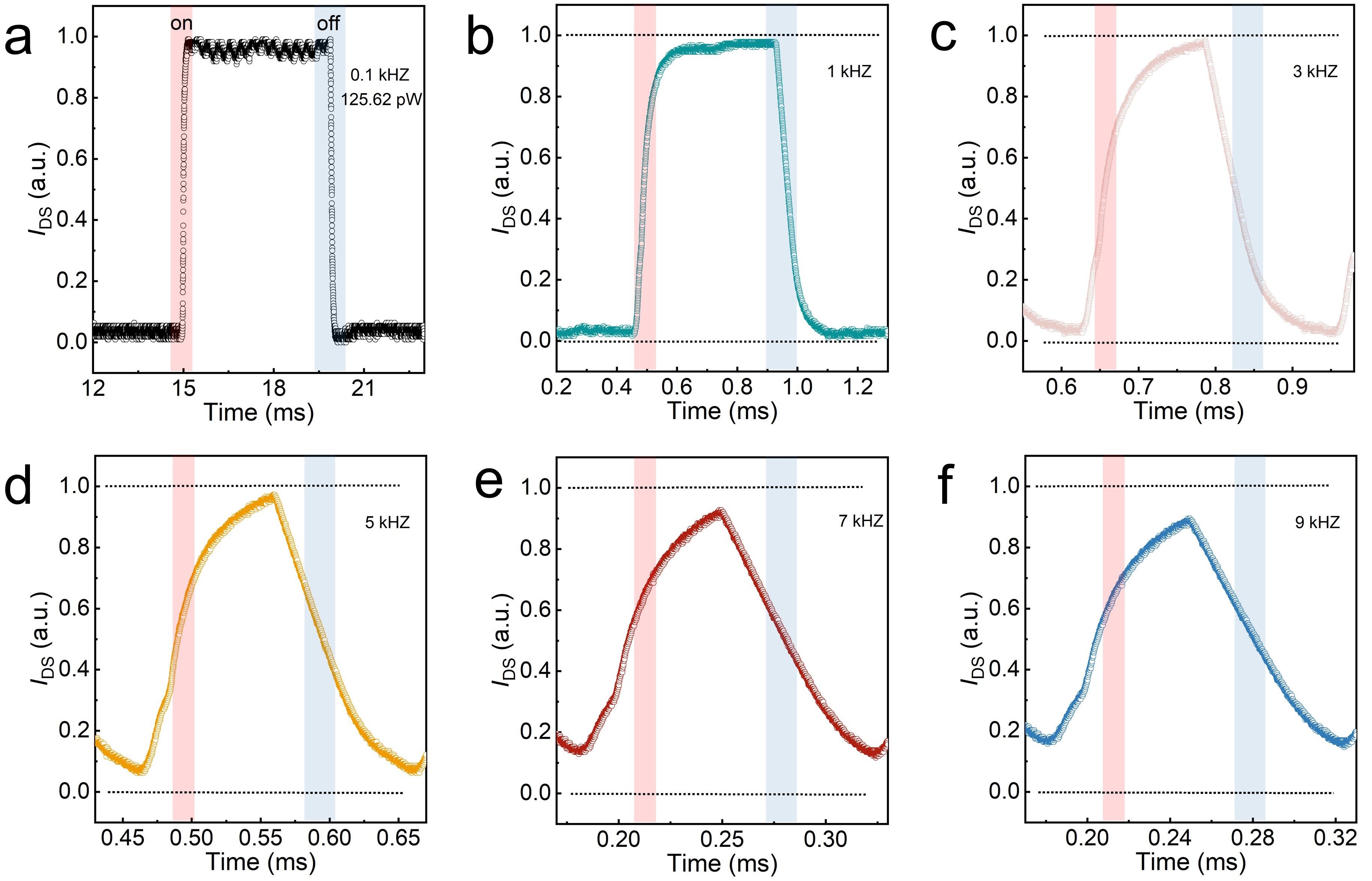


**Supplementary Figure 19.** Photo-response with different frequency at *V*_DS_ = 7.5 V and laser power of 125.62 pW.


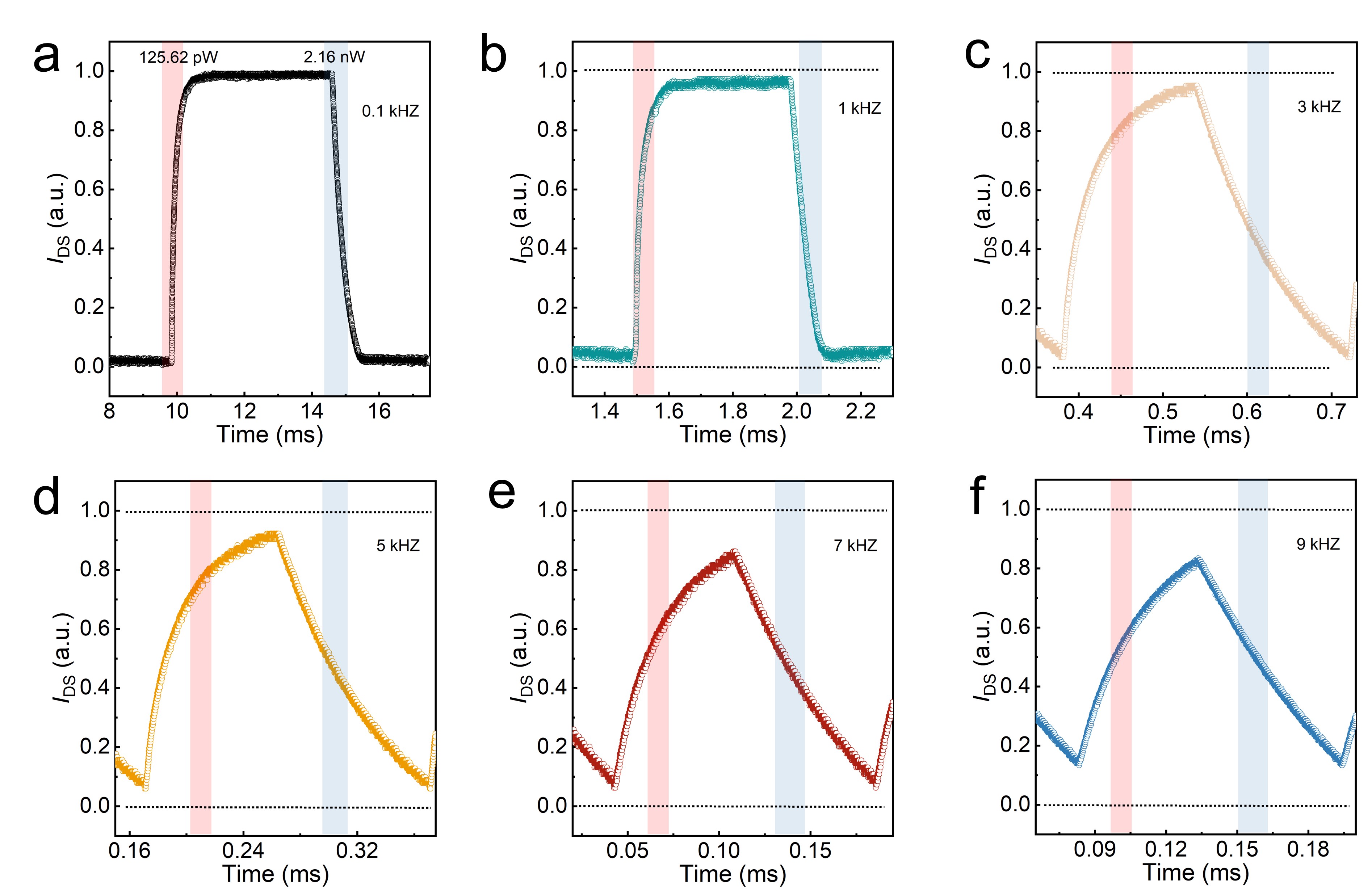


**Supplementary Figure 20.** Time-varying current by switching laser power between 125.62 and 2158.43 pW with different frequency at *V*_DS_ = 7.5 V.


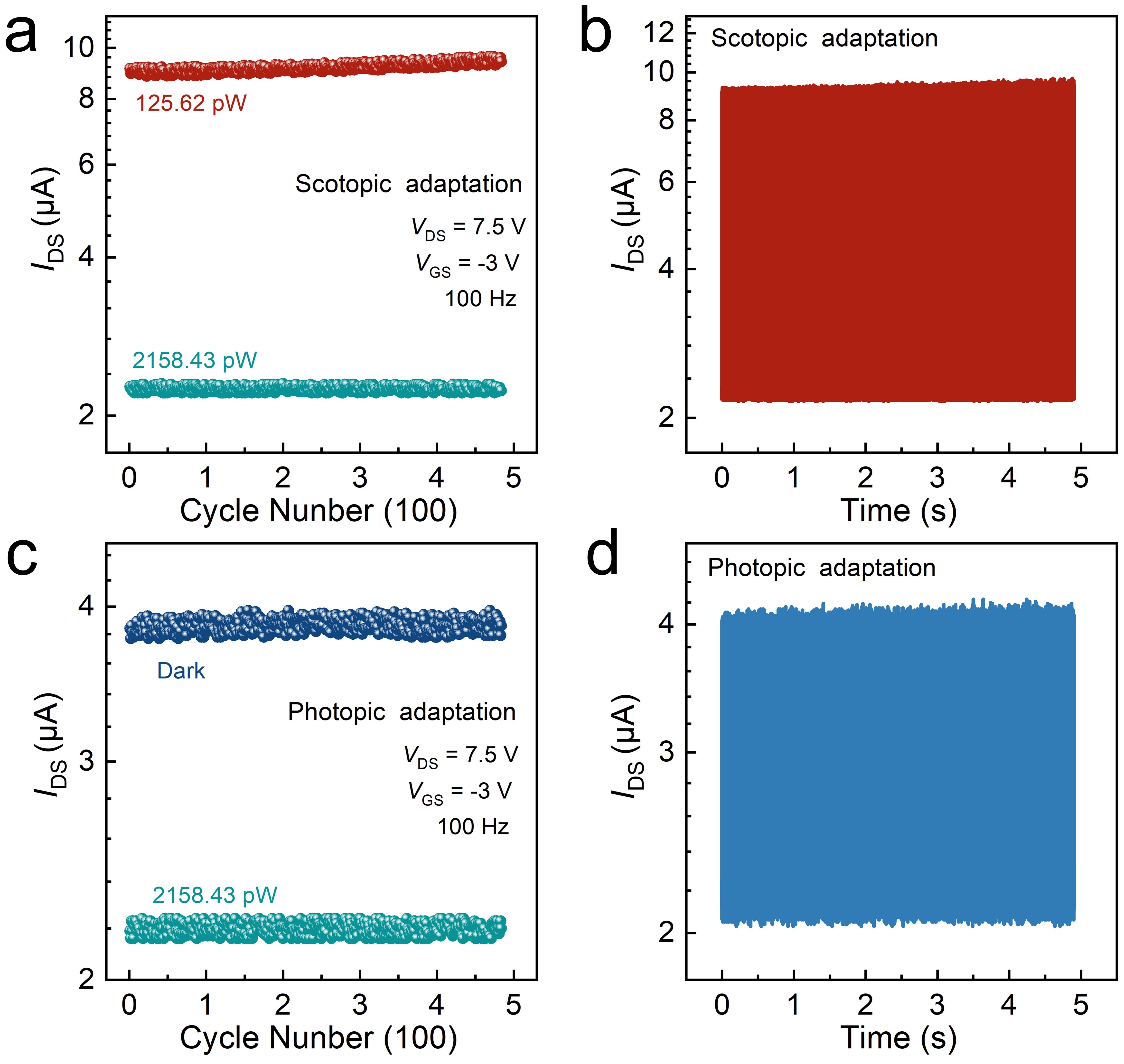


**Supplementary Figure 21.** The device operates for 500 cycles under simulated scotopic (a-b) and photopic (c-d) adaptation conditions, where **a** and **c** are extracted from **b** and **d**.


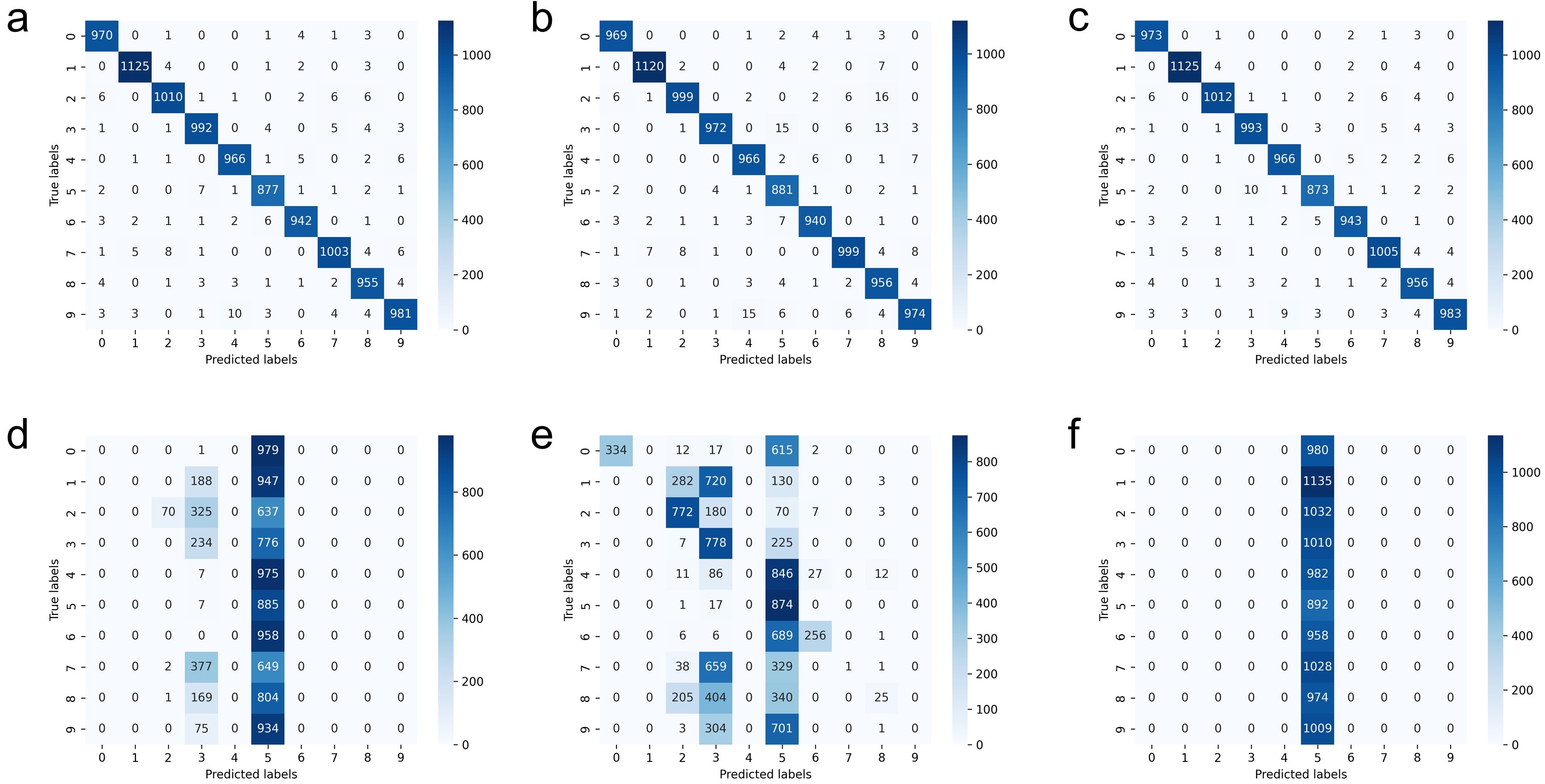


**Supplementary Figure 22.** Confusion matrix with results of 10,000 image recognition trials under (**a-c**) brightness increase or (**d-f**) decrease by 40%, 60% and 80%.

**Table 1.** Comparison of the proposed negative photoconductivity devices with previous report.

| Sensor structure | Response  time | Photoconductivity | Operating Mechanism | Operating voltage | Switching condition | Circuit motif | Ref. |
| --- | --- | --- | --- | --- | --- | --- | --- |
| MoS_2_/WSe_2_ | 108, 268 µs | Positive, Negative | Avalanche | Bias = 7.5 V, Gate = -3 V | Light intensity | Feedforward inhibition | This work |
| MoS_2_ | 120, 120 s | Positive, Negative | Trapping & De-trapping | Bias = 1 V, Gate = -3/6 V | Gate Voltage | Feedback inhibition | ^1^ |
| ReS_2_/U6N | 3 s | Negative | Trapping & De-trapping | Bias = 1 V, Gate = 40 V |  | Feedback inhibition | ^2^ |
| CsPbBr_3_/PDPP-TT | 120, 120 s | Positive, Negative | Trapping & De-trapping | Bias = -10 V, Gate = -20 V | Light intensity | Feedback inhibition | ^3^ |
| CsPbBr_3_/MoS_2_ | 2 s | Negative | Trapping & De-trapping | Bias = 0.1 V, Gate = -20 V |  | Feedback inhibition | ^4^ |
| γ-InSe nanoflake | 150 s | Negative | Photo-pyroelectric and Photo-thermoelectric | Bias = 0 V, Gate = 0 V |  | Feedback inhibition | ^5^ |
| InP /ITZO | 300/300 s | Positive, Negative | Trapping & De-trapping | Bias = 1 V, Gate = -2/3 V | Gate Voltage | Feedback inhibition | ^6^ |
| Tellurium | 20, 20 s | Positive, Negative | Trapping & De-trapping | Bias = 1 V, Gate = 0 V | Environmental gases | Feedforward inhibition | ^7^ |
| ReS_2_/h-BN/MoS_2_ | 12, 30 s | Positive, Negative | Interlayer carrier transport | Bias = 1 V, Gate = -60/60 V | Gate Voltage | Feedforward inhibition | ^8^ |

**References**

1. Liao, F. *et al.* Bioinspired in-sensor visual adaptation for accurate perception. *Nat Electron* **5**, 84–91 (2022).

2. Xie, D. *et al.* Porous Metal–Organic Framework/ReS_2_ Heterojunction Phototransistor for Polarization‐Sensitive Visual Adaptation Emulation. *Advanced Materials* **35**, 2212118 (2023).

3. Kuang, J. *et al.* Interface Defects Tuning in Polymer‐Perovskite Phototransistors for Visual Synapse and Adaptation Functions. *Adv Funct Materials* **33**, 2209502 (2023).

4. Xie, D. *et al.* Photoelectric Visual Adaptation Based on 0D‐CsPbBr_3_ ‐Quantum‐Dots/2D‐MoS_2_ Mixed‐Dimensional Heterojunction Transistor. *Adv Funct Materials* **31**, 2010655 (2021).

5. Liu, W. *et al.* Self-powered and broadband opto-sensor with bionic visual adaptation function based on multilayer γ-InSe flakes. *Light Sci Appl* **12**, 180 (2023).

6. Gao, Z. *et al.* InP Quantum Dots Tailored Oxide Thin Film Phototransistor for Bioinspired Visual Adaptation. *Adv Funct Materials* 2305959 (2023) doi:10.1002/adfm.202305959.

7. Wang, R. *et al.* Controllable Inverse Photoconductance in Semiconducting Nanowire Films. *Advanced Materials* 2204698 (2022) doi:10.1002/adma.202204698.

8. Wang, Y. *et al.* Negative Photoconductance in van der Waals Heterostructure-Based Floating Gate Phototransistor. *ACS Nano* **12**, 9513–9520 (2018).
